# Acaricidal efficacy and biochemical effects of *Cananga odorata* essential oil and three selected compounds against *Haemaphysalis doenitzi* (Acari: Ixodidae)

**DOI:** 10.1186/s13071-026-07427-9

**Published:** 2026-06-08

**Authors:** Songbo Zhang, Zhihua Gao, Boyu Yang, Xintong Liu, Chenxiao Lu, Haokun Feng, Pengxu Zhu, Zihan Liang, Ahmed H. Ghonaim, Xiaolong Yang

**Affiliations:** 1 Hebei Key Laboratory of Animal Physiology, Biochemistry and Molecular Biology, Hebei Collaborative Innovation Center for Eco-Environment, Ministry of Education Key Laboratory of Molecular and Cellular Biology, College of Life Sciences, Hebei Normal University, Shijiazhuang, 050024 China; 2 National Key Laboratory of Agricultural Microbiology, College of Veterinary Medicine, Huazhong Agricultural University, Wuhan, China; 3 Desert Research Center, Cairo, Egypt

**Keywords:** *Cananga odorata* essential oil, Lilial, Cinene, α-Amylcinnamaldehyde, Acaricidal activity, Bioactivity

## Abstract

**Background:**

Ticks are major ectoparasites that significantly impact livestock productivity worldwide. With the growing resistance to synthetic acaricides and increasing concerns about environmental and human safety, identifying effective and ecofriendly alternatives has become a pressing need. This study evaluated the fumigant and contact toxicities of ylang-ylang (*Cananga odorata*) essential oil (EO) and three plant-derived compounds, lilial, cinene, and α-amylcinnamaldehyde, against *Haemaphysalis doenitzi* and explored their underlying mechanisms of action.

**Methods:**

The acaricidal potential of *C. odorata* EO, lilial, cinene, and α-amylcinnamaldehyde against *H. doenitzi* was evaluated through in vitro (fumigation and immersion tests) and subsequent biochemical effects experiments. Surviving adult ticks treated with the semi-lethal concentration (LC_50_) were homogenized to detect GST, Na⁺/K⁺-ATPase, AchE, and CarE activities, and HDABCE1, HD-GSTa, and *HDCYP450a* transcripts were quantified via reverse-transcription quantitative polymerase chain reaction (RT–qPCR). Homology models of the three proteins were built and docked with lilial, cinene, and α-amylcinnamaldehyde to predict binding sites and affinities.

**Results:**

In the fumigation assay, *C. odorata* EO and lilial showed the highest activity with the lowest LC_50_ value (27.904 and 27.173 µg/mL, respectively), outperforming cinene and α-amylcinnamaldehyde. In the immersion test, all substances exhibited significant acaricidal activity, with α-amylcinnamaldehyde achieving 100% mortality in both nymphs and adults at 50 mg/mL after 24 h. α-Amylcinnamaldehyde LC_50_ was 4.4 mg/mL for nymphs and 10.8 mg/mL for adults. Enzyme assays indicated that lilial and *C. odorata* EO significantly inhibited GST and AchE, while cinene and α-amylcinnamaldehyde primarily induced CarE activity. Gene expression analysis further revealed that cinene and *C. odorata* EO significantly upregulated HD-GSTa and HD-CYP450ais>; lilial and *C. odorata* EO strongly elevated HDABCE1, and α-amylcinnamaldehyde markedly induced HD-CYP450ais>. Molecular docking supported these findings, showing that each compound interacts with HD-GSTa, HD-CYP450a, and HD-ABCE1 proteins through distinct binding modes, consistent with their differential mechanisms of action.

**Conclusions:**

*Cananga odorata* EO, cinene, lilial, and α-amylcinnamaldehyde potently exhibit acaricidal activity via distinct but complementary disruption of detox enzymes, neurotransmission, and efflux pumps, offering a basis for novel plant-derived acaricides.

**Graphical Abstract:**

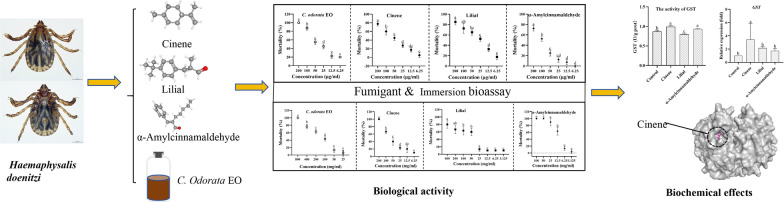

## Background

Ticks are globally distributed ectoparasites that feed exclusively on the blood of a wide range of hosts, including birds, mammals, reptiles, and amphibians [1–3]. During blood feeding, they can transmit a variety of pathogens such as viruses, rickettsiae, protozoa, and bacteria, thereby causing numerous causing various diseases in humans and animals [4]. *Haemaphysalis doenitzi (H. doenitzi)* is an obligate hematophagous tick species that infests domestic livestock, poultry, and wild birds [5]. Heavy infestations can lead to growth retardation, anemia, or even death, and this species is known to transmit pathogens such as *Rickettsia* and *Babesia*, resulting in substantial economic losses in the livestock industry [6, 7]. *H. doenitzi* is a three-host tick species that feeds on different hosts at the larval, nymph, and adult stages [8]. This feeding behavior enhances its ability to spread diseases, impacting livestock health and productivity. Controlling *H. doenitzi* is crucial for reducing economic losses and preventing zoonotic disease transmission. Following a tick bite, localized skin reactions such as redness, swelling, pain, and itching may occur; in severe cases, blisters, ulceration, or granulomas can develop, and individuals with allergic predispositions may experience anaphylactic shock [9].

Current tick control methods are primarily categorized into chemical control, biological control, and integrated pest management [10]. Chemical acaricides remain the most widely used approach owing to their rapid efficacy and low cost. However, their extensive use has led to serious drawbacks, including toxicity to nontarget organisms, environmental accumulation, and the emergence of resistance, thereby necessitating increasingly higher doses and perpetuating a resistance cycle. To overcome this challenge, this study investigated *Cananga odorata* essential oil (EO) and three plant-derived compounds commonly used in the fragrance industry, natural cinene (limonene), synthetic lilial (lysmeral), and α-amylcinnamaldehyde, as potential green alternatives to chemical acaricides. These compounds were evaluated for both fumigant and contact toxicities against *H. doenitzi*, and their molecular targets were characterized. This approach aims to identify safe, effective, and environmentally friendly acaricidal agents suitable for sustainable tick control.

Ylang-ylang (*Cananga odorata*, Annonaceae family) is native to Southeast Asia and has been introduced to regions [11]. Its essential oil possesses a rich floral fragrance, finding applications in perfumery and serving as a resource for environmentally friendly insecticides [12]. The primary constituents are sesquiterpenes, including benzyl acetate and benzyl benzoate, with their content varying slightly depending on origin and processing methods [13]. Cinene and its derivatives have wide industrial applications across the food, pharmaceutical, and cosmetic sectors [14, 15]. Cinene is a natural monoterpene predominantly found in the essential oil of *Citrus limonia* peel, characterized by a fresh citrus aroma and high volatility [16]. As a plant-defensive volatile, it exhibits repellent, fumigant, and contact activities against multiple pest species [17, 18] and has demonstrated acaricidal effects against *Rhipicephalus sanguineus* larvae [19]. Lilial is a synthetic fragrance widely incorporated into personal care and household products [20]. Previous research has shown that lilial possesses strong mosquito-repellent efficacy, rapid action, and low effective dose [21]. α-Amylcinnamaldehyde, a derivative of cinnamaldehyde, with a characteristic cinnamon aroma [22], is used in cosmetics, food, and tobacco industries [23]. Its homologs show potent contact activity against mosquito larvae [24], while (*E*)-cinnamaldehyde exhibits strong acaricidal activity against *Haemaphysalis longicornis* [25]. These have been identified as active components in blended essential oil formulations with proven efficacy and safety.

In this study, we systematically evaluated the fumigant and contact toxicities of cinene, lilial, and α-amylcinnamaldehyde against both nymphal and adult stages of *H. doenitzi*. Their effects on key detoxification and neural enzymes, glutathione *S*-transferase (GST), Na⁺/K⁺-ATPase, carboxylesterase (CarE), and acetylcholinesterase (AchE) were determined. Furthermore, reverse-transcription quantitative polymerase chain reaction (RT–qPCR) and molecular docking analyses were employed to assess target gene expression and elucidate protein-binding interactions. The findings expand the potential industrial applications of these readily available compounds and enhance understanding of their acaricidal mechanisms. Conducting systematic toxicological studies on such natural products may help overcome resistance challenges associated with conventional chemical acaricides, offering promising candidates for the development of novel, green, safe, and low-resistance tick control agents.

## Methods

### Ticks rearing

Larvae, nymphs, and adults of *H. doenitzi* used in this study were obtained from a continuously maintained laboratory colony at Hebei Normal University, located in Shijiazhuang, Hebei Province, China. New Zealand White rabbits were used as host animals for blood feeding, with ticks allowed to attach and feed on the ear pinnae. After full engorgement, ticks were immediately transferred to a thermostatic incubator maintained at 27 ± 1 °C, relative humidity (RH) ≥ 80%, and a photoperiod of 6 h light/18 h dark for molting and further development. All bioassays were performed using unfed nymphs and adults that had been maintained under these conditions for 35 days post-molt.

### Chemicals

The *C. odorata* EO was purchased from Huaxin Natural Plant Co., Ltd., Ji’an City, Jiangxi Province, China. The essential oils used are steam-distilled and blended with synthetic fragrances. They are packaged in light-protected, sealed containers and stored at 4 °C until undergoing bioactivity testing. Cinene (D807263, ≥ 95%), lilial (L812484, ≥ 97%), and α-amylcinnamaldehyde (A832228, ≥ 97%) were purchased from Shanghai McLean Biochemical Technology Co., Ltd, China. All compounds were stored at 4 °C in the dark until use. Enzyme activity assay kits, including glutathione *S*-transferase (GST; BC0355), carboxylesterase (CarE; BC0845), acetylcholinesterase (AchE; BC2025), and Na⁺/K⁺-ATPase (BC0065), were obtained from Beijing Solarbio Science and Technology Co., Ltd (China).

### Fumigant bioassay

The fumigant toxicity of the compounds was evaluated using the glass bottle (7 mL) fumigation method, similar to that used in Bin Yan’s 2024 study [26]. Each reagent (*C. odorata* EO, cinene, lilial, and α-amylcinnamaldehyde) was dissolved in 2% acetone to prepare serial concentrations of 200, 100, 50, 25, 12.5, and 6.25 µg/mL. Using a micropipette, dispense 30 μL of each solution onto a piece of filter paper fixed at the inner surface of the bottle cap. The samples were labeled, covered with aluminum foil, and left in a well-ventilated area for 20 min to allow solvent evaporation. A total of ten unfed nymph ticks were then placed into each bottle, which was sealed immediately with perforated aluminum foil to allow air exchange. The bottles were incubated at 27 ± 1 °C, RH ≥ 80%, and a 6 h light/18 h dark photoperiod for 24 h. Mortality was assessed on the basis of retracted limbs and the absence of movement upon gentle probing with a fine brush. Each treatment was replicated three times, and a 2% acetone solution served as the control.

### In vitro immersion test

Contact toxicity of the compounds against ticks was evaluated using the in vitro immersion bioassay [27]. Test solutions of *C. odorata* EO, cinene, lilial, and α-amylcinnamaldehyde were prepared in 2% Tween 80 at serial concentrations of 800, 400, 200, 100, 50, 25, 12.5, 6.25, and 3.125 mg/mL. For each replicate, ten unfed nymphs or adults were placed into a 1.5-mL microcentrifuge (EP) tube, followed by the addition of 1 mL of the test solution. Ticks were immersed for 5 min, after which the solution was discarded. Ticks were then gently transferred onto filter paper to remove excess liquid and placed into clean tubes.

All samples were incubated at 27 ± 1 °C, RH ≥ 80%, and a 6 h light/18 h dark photoperiod for 24 h. Mortality was determined by curled appendages and lack of touch response. Mortality rate (%) = (number of dead ticks/total number of ticks) × 100%. Each treatment included three replicates, with 2% Tween 80 serving as the blank control.

### Enzyme activity assays

Enzyme activities were assessed in unfed adult *H. doenitzi* ticks exposed to LC_50_ concentrations of *C. odorata* EO, cinene, lilial, and α-amylcinnamaldehyde for 5 min, followed by incubation at 27 ± 1 °C and ≥ 80% RH for 24 h. Control ticks were treated with 2% Tween 80 only, and enzyme activities were measured under the same conditions as the experimental groups to establish baseline enzyme activity levels. The ticks that had survived were then selected for further analysis. To standardize samples for enzyme activity assays, we ensured consistent amounts of tick material were used, adhering to established protocols for tick enzyme analysis. Assays were conducted at 37 °C according to standard protocols, with one unit (U) defined as the amount of enzyme catalyzing specific substrate reactions per gram tissue per minute. GST activity was measured by the conjugation of 1-chloro-2,4-dinitrobenzene (CDNB) with glutathione (GSH). Carboxylesterase (CarE) and acetylcholinesterase (AchE) activities were determined by changes in absorbance at 340 nm. Na⁺/K⁺-ATPase activity was assayed by the release of inorganic phosphate (Pi). Enzyme activities (U/g) were calculated as: enzyme activity = (moles of product formed/*W* × *t*), where *W* is tissue mass (g) and *t* is reaction time (min for GST, CarE, AchE; h for Na⁺/K⁺-ATPase). For detailed procedures, we refer to established methods in literature [27].

### Gene expression analysis

Samples for real-time quantitative PCR (RT–qPCR) were the same as those used for enzyme activity assays. A total of 30 unfed adult ticks that survived treatment with *C. odorata* EO, cinene, lilial, and α-amylcinnamaldehyde at sublethal concentrations for 5 min were incubated for 24 h at 27 ± 1 °C and RH ≥ 80%, rapidly frozen in liquid nitrogen, and stored at −80 °C for total RNA extraction and subsequent gene expression analysis. The sample comprised three biological replicates, each with ten ticks. Control ticks were treated with 2% Tween 80 only and subjected to gene expression analysis under identical conditions as the experimental groups to establish a suitable baseline for normalization. Total RNA was extracted using the TRIzol method, and residual genomic DNA was removed using DNase I. First-strand complementary DNA (cDNA) was synthesized by reverse transcription using the EasyScript^®^ One-Step gDNA Removal and cDNA Synthesis SuperMix kit (Beijing Quanshijin) according to the manufacturer’s instructions.

SYBR Green I RT–qPCR was performed to quantify the expression of three target genes: HDABCE1 (ABC transporter), HD-GSTa (glutathione *S*-transferase), and HD-CYP450ais> (cytochrome P450), using *β-actin* as the internal reference gene. Relative expression levels were calculated using the 2^−ΔΔCt^ method based on Ct values and subjected to statistical analysis. All primers were designed according to previously reported sequences [27].

### Molecular docking

Molecular docking simulations were performed using AutoDock software. The crystal structures of the target proteins were modeled using SWISS-MODEL based on the following GenBank accession numbers: HDABCE1 (XOD50192.1), HD-GSTa (PQ657472.1), and HD-CYP450 (PP962429.1). Hydrogen atoms and side chains were added to the protein structures, water molecules within 5 Å of the ligands were removed, and polar hydrogen atoms were added using AutoDockTools 1.5.7. The three-dimensional structures of the ligands (cinene, lilial, and α-amylcinnamaldehyde) were obtained from PubChem and saved in PDB format.

Docking grids were centered on the predicted active sites to ensure full coverage. The conformation with the lowest binding energy was selected as the optimal docking pose. The resulting protein–ligand interactions were visualized and analyzed using PyMOL, and key parameters, including binding energy (kcal/mol) and hydrogen bond interactions, were recorded.

### Statistical analysis

All data were statistically analyzed using SPSS 26.0. Differences in mortality among groups were assessed using one-way analysis of variance (ANOVA), followed by Tukey’s honestly significant difference (HSD) test for multiple comparisons (*α* = 0.05). LC₅₀ and LC_90_ values were estimated by Probit regression analysis with 95% confidence intervals. Comparisons between control and treatment groups for enzyme activity and RT–qPCR data were performed using independent samples *t*-tests, while differences among multiple treatments were further analyzed using Tukey’s HSD test.

## Results

### Fumigant toxicity

**Fig. 1:**
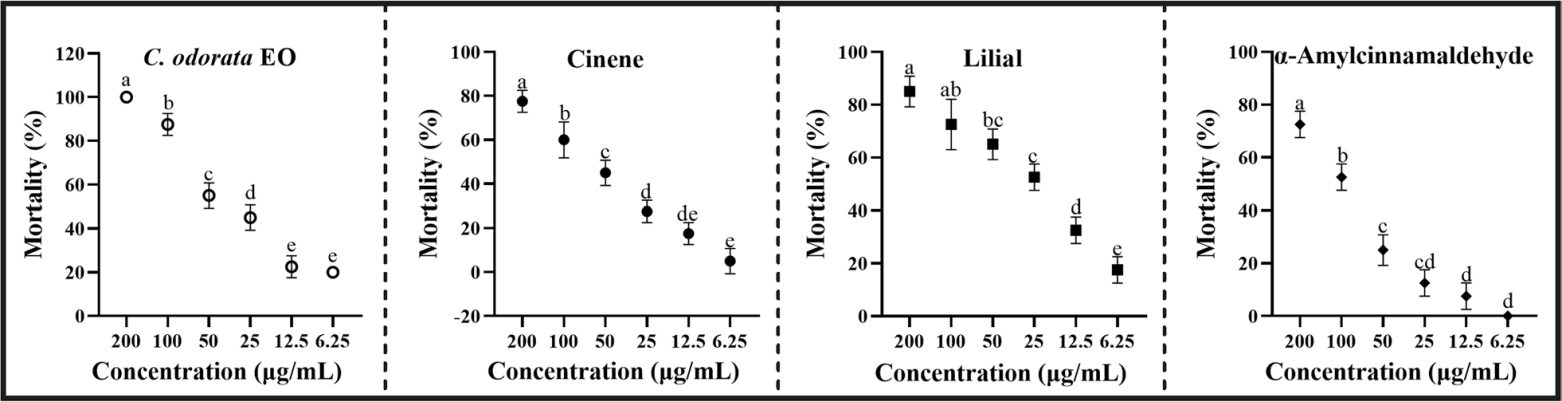
Fumigant toxicity of *C. odorata* EO, cinene, lilial, and α-amylcinnamaldehyde against *H. doenitzi* unfed nymphs after 24 h. Different letters above bars indicate significant differences among treatments based on Tukey’s multiple comparison test (*P* < 0.05)

As shown in Fig. 1, the mortality rate of ticks increased in a dose-dependent manner with increasing concentrations of *C. odorata* EO, cinene, lilial, and α-amylcinnamaldehyde. At 200 µg/mL, there was no significant difference in fumigant activity among the four treatments, with mortality rates approaching 80% across all compounds. To further quantify their fumigant efficacy, toxicity parameters were determined (Table 1). The LC_50_ values for *C. odorata* EO, cinene, lilial, and α-amylcinnamaldehyde after 24 h were 27.904, 63.146, 27.173, and 98.123 µg/mL, respectively, while the LC_90_ values were 142.440, 446.457, 309.816, and 468.876 µg/mL, respectively. Among them, *C. odorata* EO and lilial exhibited the lowest LC_50_ values, indicating the highest fumigant potency. Therefore, *C. odorata* EO and lilial achieved comparable lethality to cinene and α-amylcinnamaldehyde at significantly lower concentrations.

**Table 1:** Fumigant activity of *C. odorata* EO, cinene, lilial, and α-amylcinnamaldehyde against unfed nymphs *H. doenitzi*

Treatment	*N*	DF	Regression eq. (*Y* =)	LC_50_ (95% CI)	LC_90_ (95% CI)	*χ*^2^	Slope ± SE
*C. odorata *EO	240	5	0.0041*x* + 0.2784	27.904^b^ (21.640–35.428)	142.440^b^ (99.399–244.042)	88.283	1.810 ± 0.219
Cinene	240	5	0.0034*x* + 0.1623	63.146^a^ (47.913–87.231)	446.457^a^ (261.148–1077.610)	62.515	1.509 ± 0.205
Liial	240	5	0.0029*x* + 0.3506	27.173^b^ (18.507–37.803)	309.816^a^ (173.189–857.200)	51.894	1.212 ± 0.187
α-Amylcinnamaldehyde	240	5	0.0037*x* + 0.0386	98.123^a^ (76.525–134.451)	468.876^a^ (293.105–1002.270)	91.499	1.887 ± 0.249

*LC* lethal concentration (µg/mL), *CI* confidence interval, *DF* degree of freedom, *χ*^*2*^ chi-squared value. The different letters of each column are significantly different at *P* < 0.05

### In vitro immersion test

**Fig. 2:**
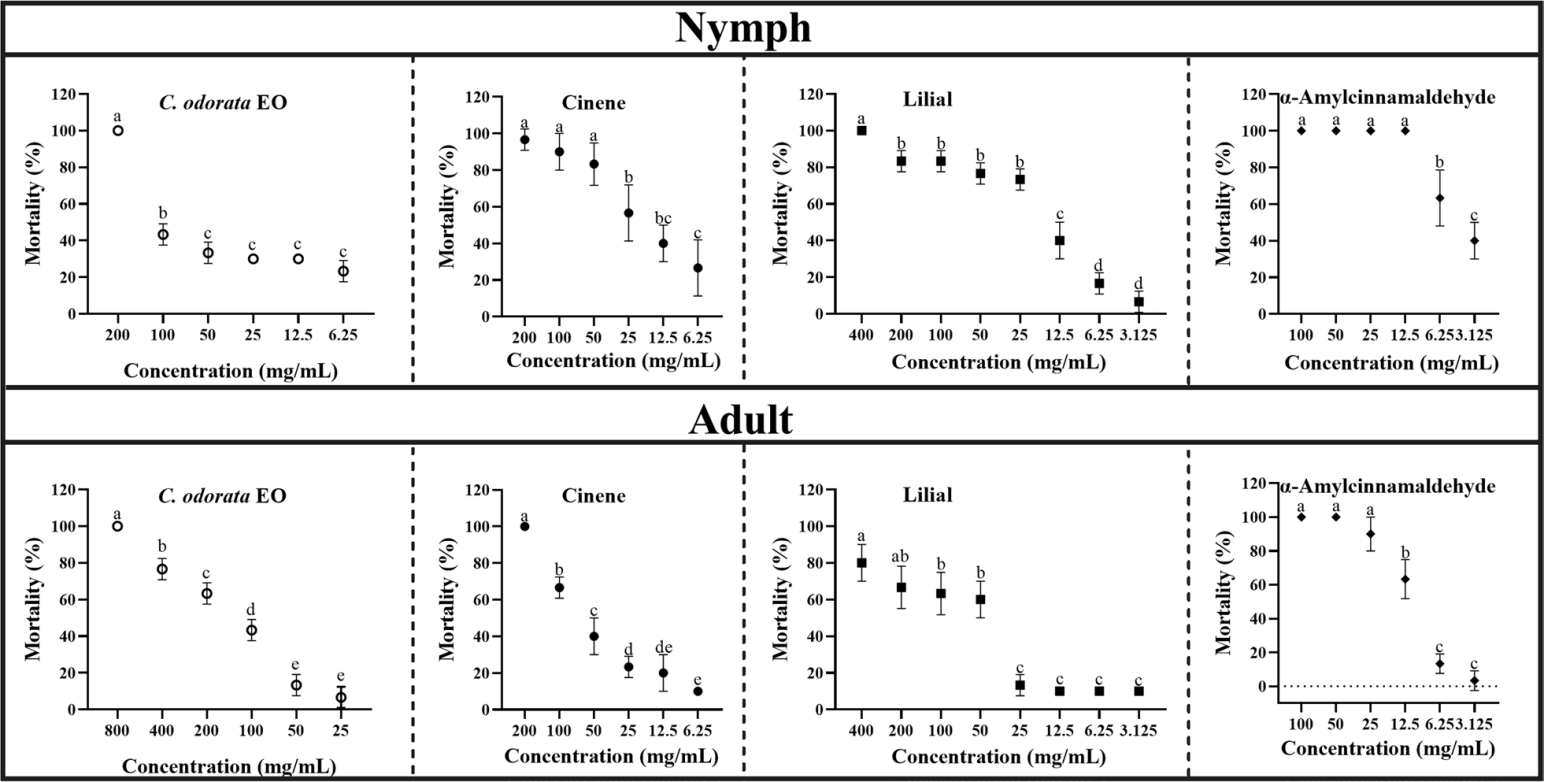
In vitro immersion test of *C. odorata* EO, cinene, lilial, and α-amylcinnamaldehyde against *H. doenitzi* unfed nymphs and adults after 24 h treatment. Different letters above bars indicate significant differences among treatments based on Tukey’s multiple comparison test (*P* < 0.05)

The lethal dynamics of the four reagents against adult and nymph ticks, *H. doenitzi*, in the immersion bioassay are shown in Fig. 2. The highest contact toxicity was observed for α-amylcinnamaldehyde on both developmental stages, achieving 100% mortality at 50 mg/mL. Cinene and lilial also demonstrated significant acaricidal activity: cinene caused 100% mortality in both adults and nymphs at 200 mg/mL, while lilial achieved 100% mortality in nymphs and 80% mortality in adults at 400 mg/mL. *C. odorata* EO exhibited the poorest acaricidal activity. The corresponding toxicity parameters (Table 2) revealed LC_50_/LC_90_ values for nymphs as follows: *C. odorata* EO (30.108/188.981 mg/mL), cinene (16.532/95.900 mg/mL), lilial (19.205/144.092 mg/mL), and α-amylcinnamaldehyde (4.397/9.122 mg/mL). For adults, the LC_50_/LC_90_ values were 139.544/513.069, 46.588/232.495, 72.388/900.762, and 10.781/23.537 mg/mL, respectively. All reagents exhibited significantly higher toxicity against nymphs than adults. Among them, α-amylcinnamaldehyde demonstrated the lowest lethal concentration and the most pronounced acaricidal efficacy, confirming its superior contact toxicity profile.

**Table 2:** Toxicity of *C. odorata* EO, cinene, lilial, and α-amylcinnamaldehyde against unfed nymphs and adults *H. doenitzi*

Stage	Treatment	*N*	DF	Regression eq. (*Y* =)	LC_50_ (95%CI)	LC_90_ (95%CI)	*χ*^2^	Slope ± SE
Unfed nymphs	*C. odorata* EO	180	5	0.0082*x* + 0.1187	30.108^a^ (21.488–45.220)	188.981^a^ (103.743–566.443)	76.308	1.606 ± 0.239
	Cinene	180	5	0.0032*x* + 0.4461	16.532^a^ (11.439–22.230)	95.900^a^ (64.281–181.680)	54.817	1.679 ± 0.244
	Liial	240	7	0.001*x* + 0.627	19.205^a^ (13.817–25.884)	144.092^a^ (95.023–261.430)	103.333	1.464 ± 0.166
	α-Amylcinnamaldehyde	180	5	0.004*x* + 0.7073	4.397^b^ (3.335–5.331)	9.122^b^ (7.292–13.872)	75.182	4.043 ± 0.826
Unfed adults	*C. odorata* EO	180	5	0.0011*x* + 0.2138	139.544^a^ (109.248–177.024)	513.069^a^ (368.735–848.383)	76.638	2.266 ± 0.294
	Cinene	180	5	0.0045*x* + 0.1348	46.588^b^ (35.616–62.596)	232.495^a^ (149.546–468.252)	71.131	1.836 ± 0.243
	Liial	240	7	0.0022*x* + 0.3303	72.388^a^ (50.762–109.067)	900.762^a^ (464.035–2548.846)	83.871	1.170 ± 0.146
	α-Amylcinnamaldehyde	180	5	0.0087*x* + 0.3303	10.781^c^ (8.944–12.911)	23.537^b^ (18.777–33.296)	120.353	3.779 ± 0.530

*N* sample size, *LC* lethal concentration (mg/mL), *CI* confidence interval, *DF* degree of freedom, *χ*^*2*^ chi-squared value. In each stage, the data followed with different letters of each column are significantly different at *P* < 0.05

### Enzyme activity

**Fig. 3:**
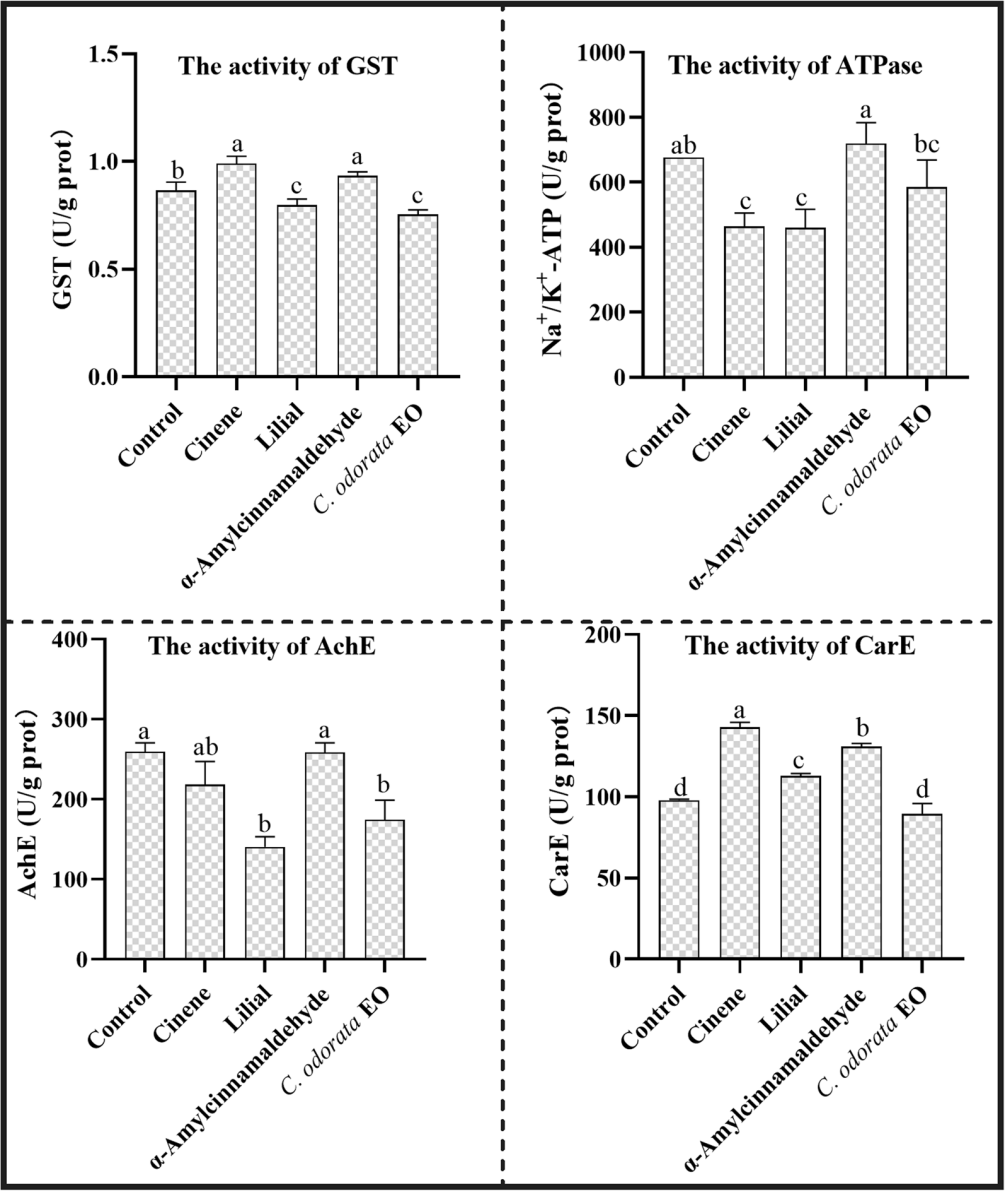
Effects of cinene, lilial, α-amylcinnamaldehyde, and *C. odorata* EO on the activities of Na⁺/K⁺-ATPase (ATPase), glutathione *S*-transferase (GST), acetylcholinesterase (AchE), and carboxylesterase (CarE) in *H. doenitzi* adults after 24 h LC₅₀ treatment. Different letters above bars indicate significant differences among treatments based on Tukey’s multiple comparison test (*P* < 0.05)

Following 24 h LC_50_ treatment, *C. odorata* EO, cinene, lilial, and α-amylcinnamaldehyde induced differential effects on four key detoxification and neuroregulatory enzymes (Fig. 3). Lilial significantly inhibited GST, Na⁺/K⁺-ATPase, and AchE, reducing their activities to 0.92-, 0.68-, and 0.54-fold of control levels, respectively (*P* < 0.05). In contrast, cinene and α-amylcinnamaldehyde primarily induced GST and CarE activities (up to 1.46-fold, *P* < 0.05), while α-amylcinnamaldehyde had no significant effect on Na⁺/K⁺-ATPase or AchE. Essential oil components are the most complex, significantly inhibiting GST and AchE activity (*P* < 0.05). This suggests that they exert multitarget acaricidal effects by interacting with multiple enzyme systems involved in detoxification and neural regulation.

### Gene expression analysis by RT–qPCR

**Fig. 4:**
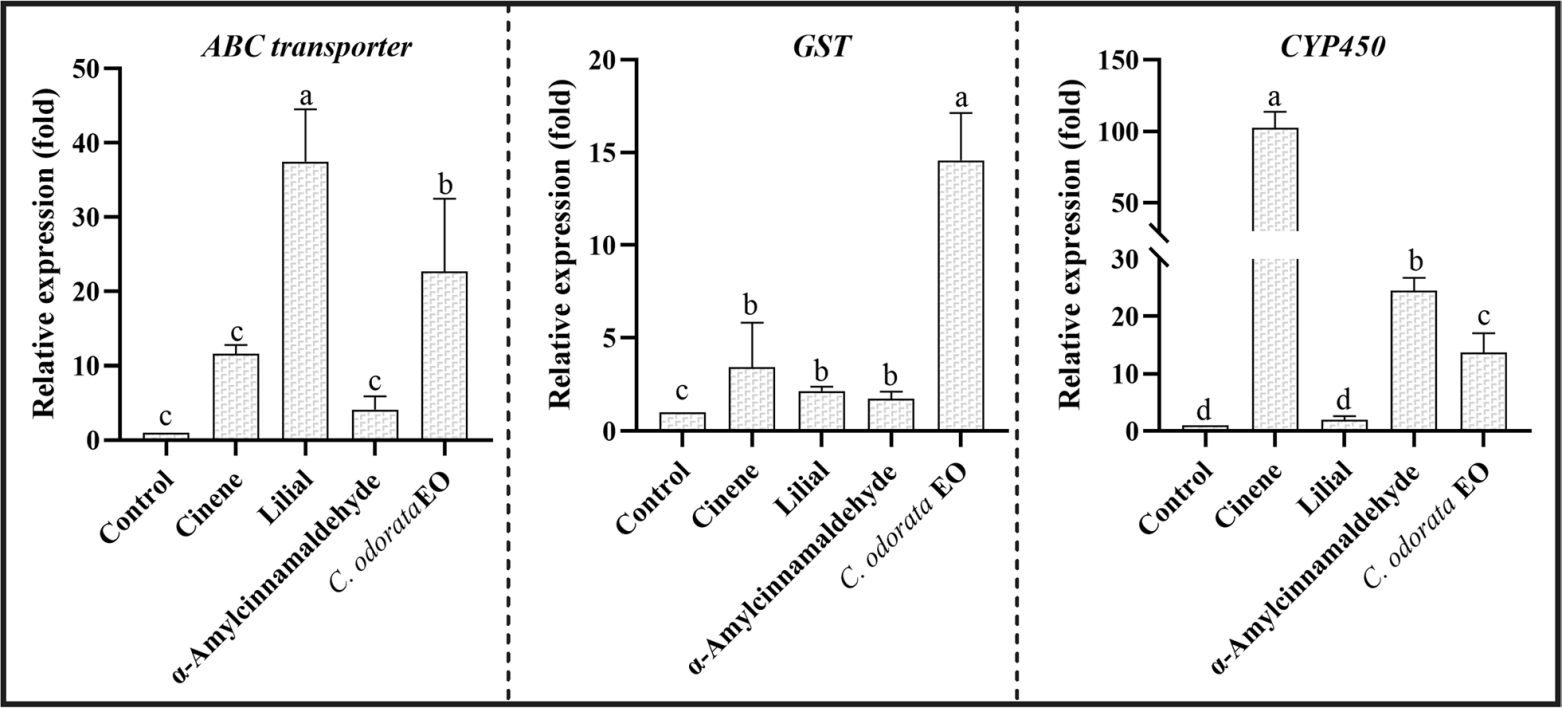
Relative mRNA expression levels of HDABCE1 (ABC transporter), HD-GSTa (GST), and HD-CYP450ais> (CYP450) genes in *H. doenitzi* adults following 24 h LC_50_ treatment with cinene, lilial, α-amylcinnamaldehyde, and *C. odorata* EO. Different letters above bars indicate significant differences among treatments based on Tukey’s multiple comparison test (*P* < 0.05)

The expression levels of HDABCE1 (ABC transporter), HD-GSTa (GST), and HD-CYP450ais> (CYP450) genes in unfed adult ticks after 24 h treatment at the LC_50_ were assessed by RT–qPCR (Fig. 4). The four reagents exhibited distinct regulatory effects on detoxification-related genes. Lilial, cinene, and *C. odorata* EO significantly upregulated HDABCE1 expression, reaching 38.47-, 12.54-, and 22.66-fold of control levels, respectively (*P* < 0.05), while α-amylcinnamaldehyde showed no significant effect. HD-GSTa expression was significantly elevated in the cinene and *C. odorata* EO treated group (3.45- and 14.58-fold increase, *P* < 0.05), with no notable changes observed for lilial or α-amylcinnamaldehyde compared to the control. In contrast, HD-CYP450ais> expression was strongly induced by cinene, α-amylcinnamaldehyde, and *C. odorata* EO, increasing to 102.37-, 24.53, and 13.72-fold of control levels (*P* < 0.05), whereas lilial showed no significant difference. Our findings indicate that cinene activates GST- and CYP450-related detoxification pathways, α-amylcinnamaldehyde primarily induces CYP450, and lilial enhances HDABCE1 expression. These transcriptional changes suggest that the four reagents may exert acaricidal effects through distinct mechanisms involving detoxification enzyme systems. However, further research is needed to fully understand the molecular mechanisms underlying these effects.

### Molecular docking

**Fig. 5:**
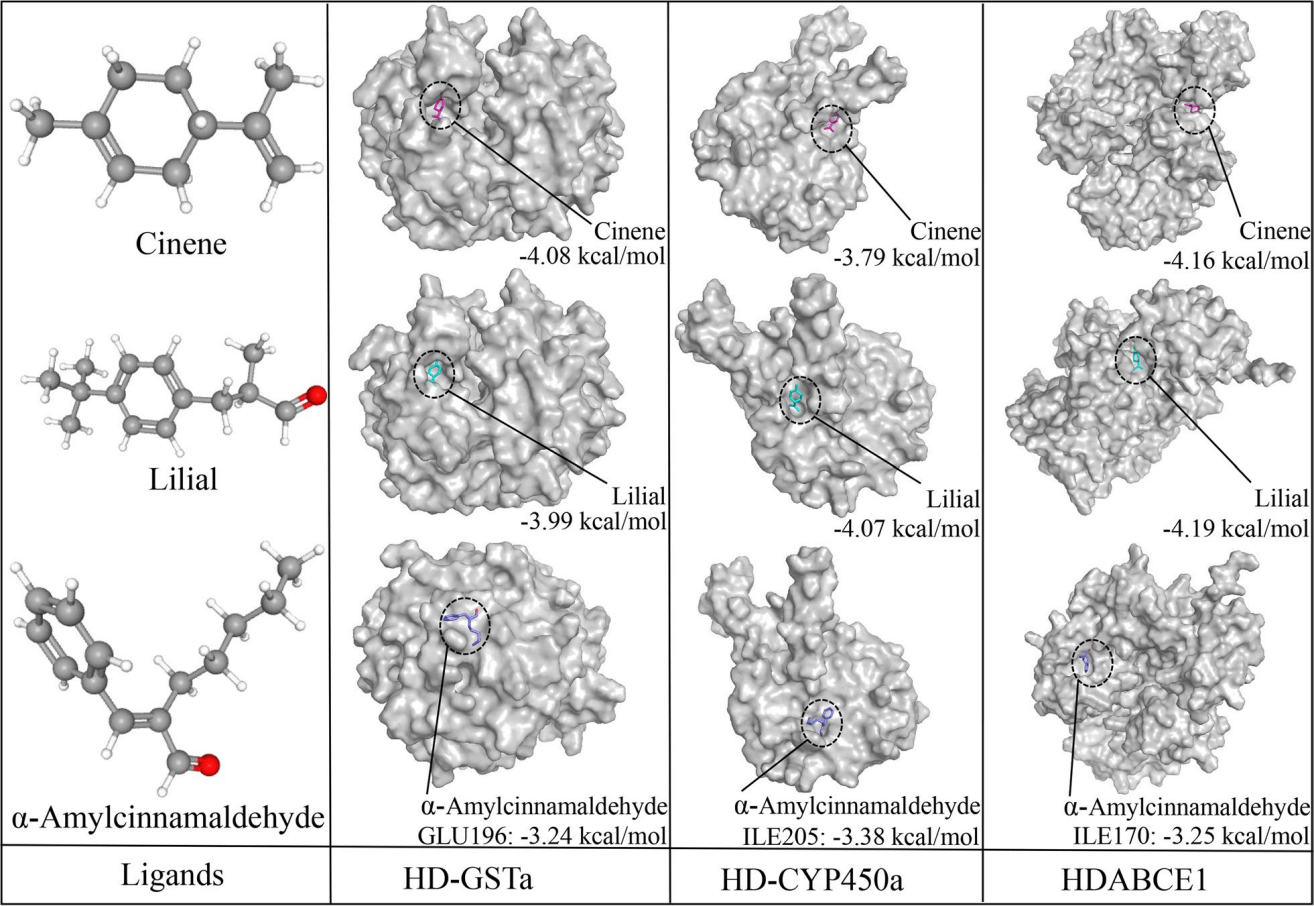
Molecular docking visualization showing the distinct binding modes of cinene, lilial, and α-amylcinnamaldehyde with *H. doenitzi* HD-GSTa, HD-CYP450a, and HDABCE1 proteins, illustrating compound-specific interaction sites and hydrogen bonding patterns

On the basis of the gene expression results, molecular docking was conducted to elucidate whether cinene, lilial, and α-amylcinnamaldehyde interact with target proteins through distinct mechanisms. The three-dimensional structural models of HD-GSTa, HD-CYP450, and HDABCE1 are illustrated in Fig. 5. Docking simulations revealed that each compound exhibited unique binding affinities and interaction profiles with the three target proteins.

For cinene, the binding energies with HD-GSTa, HD-CYP450, and HDABCE1 were −4.08, −3.79, and −4.16 kcal/mol, respectively. Lilial demonstrated comparable affinities, with docking energies of −3.99, −4.07, and −4.19 kcal/mol. In contrast, α-amylcinnamaldehyde displayed slightly weaker binding energies of −3.24, −3.38, and −3.25 kcal/mol, forming stable hydrogen bonds with key residues *GLU196*, *ILE205*, and *ILE170*. Overall, the three ligands exhibited distinct binding energies and interaction modes with the same target proteins.

## Discussion

This study represents the first systematic evaluation of the fumigant and contact toxicity of cinene, lilial, and α-amylcinnamaldehyde against *H. doenitzi*, integrating enzyme activity, gene expression, and molecular docking data to reveal their multitarget action mechanisms. Fumigation experiments demonstrated that the LC_50_ values of cinene, lilial, α-amylcinnamaldehyde, and *C. odorata* EO against nymphs within 24 h were 63.146, 27.173, 98.123, and 27.904 µg/mL, respectively. Previous research has reported that cinene exhibits excellent fumigant toxicity against *Tribolium confusum* [28], while *Piper nigrum* L. fruit essential oil, rich in cinene, achieved a fumigant LC_50_ of 13.72 mg/L air for the stable fly [29]. In contrast, toxicological evaluations of lilial, α-amylcinnamaldehyde, and *C. odorata* EO in other insects remain limited. All nymphs of *Ixodes ricinus* exposed to 0.4 μL/cm^2^ of *C. odorata* EO for 2 and 2.5 consecutive hours died [30]. Among the tested reagents, lilial and *C. odorata* EO exhibited the highest potency, combining rapid action with a clear dose advantage. This strong fumigant activity of *C. odorata* EO can be attributed to its complex chemical composition, primarily benzyl acetate, benzyl benzoate, and linalool, which may act synergistically in the vapor phase to disrupt tick nervous and detoxification systems [13]. However, fumigation primarily reflects acute knockdown effects; thus, the immersion method was employed to allow the solution to penetrate the body wall directly, more accurately demonstrating sustained acaricidal efficacy and providing a foundation for formulation and field application [31].

The contact toxicity assays revealed that cinene, lilial, α-amylcinnamaldehyde, and *C. odorata* EO exhibited strong acaricidal activity against both nymphs and adults *H. doenitzi*. The LC₅₀ values against adults were 46.588, 72.388, 10.781, and 139.544 mg/mL, respectively, which were significantly higher than those for nymphs (16.532, 19.205, 4.397, and 30.108 mg/mL, respectively). In comparison, limonene exhibited LC₅₀ values of 1.53% and 6.81% against *Rhipicephalus annulatus* and *Rhipicephalus sanguineus* larvae, respectively [32], while limonene (LC_50_ = 22.0%) showed the weakest acaricidal efficacy against *Rhipicephalus microplus* [33]. Although lilial demonstrated weaker acaricidal activity than cinene, it has been reported as a plant-derived repellent against mosquitoes [21]. Notably, α-amylcinnamaldehyde displayed the strongest lethal effect against *H. doenitzi*, consistent with findings that Niemann–Pick type C2 proteins in *Rhipicephalus linnaei* can detect its chemical signal [34]. Similarly, (*E*)-cinnamaldehyde showed LC_50_ values of 3.15 and 16.93 mg/mL against *H. longicornis* larvae and nymphs, respectively [25]. When *R. microplus* adults were immersed in *C. odorata* EO, the 7 and 14 day LC_50_ values were 25 and 40 μL/mL, respectively [35]. Given that adult ticks are critical for population reproduction, subsequent mechanistic studies will uniformly employ LC₅₀ doses for adult ticks to elucidate precise molecular targets that disrupt detoxification systems and reproductive processes, thereby supporting the design of green pasture acaricide formulations.

Beyond acute toxicity, this study provides in-depth insights into how these compounds modulate detoxification, neurotransmission, and oxidative stress-associated enzymes. Both AchE and CarE belong to the hydrolase enzyme class. AchE terminates nerve impulses via acetylcholine hydrolysis; its inhibition induces sustained paralysis [36, 37]. CarE, in contrast, catalyzes carboxylester bond cleavage, contributing to the metabolic detoxification of esterified xenobiotics [38]. GST, a key Phase II detoxification enzyme, conjugates reduced glutathione to electrophilic substrates, facilitating the excretion of toxic compounds [39]. Na⁺/K⁺-ATPase, which maintains membrane potential and osmotic balance via ATP hydrolysis-driven ion transport, is frequently a secondary target of insecticides [40]. All three plant-derived compounds significantly induced CarE activity, consistent with previous reports showing pesticide-induced CarE stimulation in insect larvae [41]. Lilial concurrently inhibited GST, Na⁺/K⁺-ATPase, and AchE while significantly upregulating CarE. *C. odorata* EO inhibits GST and AchE activity, suggesting it disrupts energy metabolism and cholinergic signaling via a dual mechanism of detoxification enzyme induction and neurotarget inhibition, leading to tick paralysis. This mechanism parallels findings in *Spodoptera frugiperda*, where matrine inhibits AchE while enhancing CarE detoxification [42]. Similarly, cadmium exposure markedly increased GST activity in *Nilaparvata lugens* [43]. In this study, α-amylcinnamaldehyde also induced GST activity, consistent with reports of elevated GST levels in bees exposed to cinnamaldehyde, suggesting that its primary mechanism involves phase II enzyme induction to reduce reactive oxygen species and overall toxic load [44]. Cinene, which induced GST expression while inhibiting Na⁺/K⁺-ATPase, may gradually weaken tick resistance mechanisms [45]. Cinene, lilial, and α-amylcinnamaldehyde disrupt ticks’ detoxification, neural signaling, and energy homeostasis through activation of CarE and GST and selective inhibition of AchE or Na⁺/K⁺-ATPase, establishing a robust molecular toxicological foundation for developing ecofriendly, high-efficacy, and low-resistance acaricides.

RT–qPCR analysis revealed that cinene, *C. odorata* EO, and α-amylcinnamaldehyde significantly upregulated CYP450 gene expression in *H. doenitzi*, consistent with the role of cytochrome P450 monooxygenases as central mediators of xenobiotic metabolism [46]. Exposure to chlorpyrifos, cypermethrin, and deltamethrin has been shown to induce *CYP6AE70* expression in *Spodoptera exigua*, with overexpression enhancing tolerance to these insecticides [47], while silencing of the *AccCYP6k1* gene reduces pesticide tolerance and disrupts detoxification and antioxidant processes in *Apis cerana* [48]. Molecular docking demonstrated that cinene and α-amylcinnamaldehyde bound to the tick CYP450 protein with energies of −3.79 and −3.38 kcal/mol, respectively, with the latter forming a hydrogen bond with GLU196, supporting its superior toxic potency [49]. Similar molecular docking validation has been reported for CYP450 in *Tribolium castaneum* [50]. The cinene-induced upregulation of GST genes corresponded to enhanced enzyme activity, providing molecular evidence of increased detoxification capacity [51], consistent with studies showing that *SlGSTe11* overexpression enhances *Spodoptera litura* tolerance to cyantraniliprole and nicotine [52]. Molecular docking further revealed cinene binds the GST protein of *H. doenitzi* with −4.08 kcal/mol affinity, indicating stable active-site occupation that promotes Phase II detoxification reactions and toxin clearance [53].

Additionally, cinene, *C. odorata* EO, and lilial significantly upregulated ABC transporter gene expression, suggesting joint activation of the efflux pump defense systems [54]. The upregulation of ABC transporters has been associated with enhanced exogenous toxin efflux in *Rhipicephalus microplus* under ivermectin stress [55], contributing to overall detoxification capacity [56]. Molecular docking confirmed strong interactions of cinene, lilial, and α-amylcinnamaldehyde with the ABC transporter (−4.16, −4.19, and −3.25 kcal/mol, respectively), with lilial exhibiting the highest binding affinity, consistent with its gene expression pattern. All three compounds formed hydrogen bonds with key residues, further supporting the superior acaricidal potential of cinnamaldehyde derivatives [34, 57].

This study was conducted under controlled laboratory conditions with a 24 h exposure period. This may not fully reflect the conditions in the field. The long-term effects, sublethal impacts on tick reproduction and nontarget safety remain to be investigated. Future research will focus on assessing field efficacy, monitoring resistance, and optimizing formulations to develop practical, environmentally sustainable tick control strategies.

## Conclusions

This study demonstrates that *C. odorata* EO, cinene, lilial, and α-amylcinnamaldehyde can simultaneously exert fumigant and contact toxicity against *Haemaphysalis doenitzi*, with α-amylcinnamaldehyde showing the strongest contact activity. Integrated analyses of enzyme activity, gene expression, and molecular docking reveal that each compound exerts a multitarget mode of action involving detoxification disruption, neurotoxicity, and inhibition of transport pumps. Collectively, these results demonstrate that *C. odorata* EO, cinene, lilial, and α-amylcinnamaldehyde achieve potent, low-residue tick control through differentiated enzyme-targeted mechanisms, offering promising candidates for next-generation green acaricides.

## Data Availability

Data supporting the main conclusions of this study are included in the manuscript.
